# Causes and Management of Cutaneous Adverse Drug Reactions: A Comprehensive Review

**DOI:** 10.7759/cureus.55318

**Published:** 2024-03-01

**Authors:** Arsh Sutaria, Shobha Rawlani, Amita H Sutaria

**Affiliations:** 1 Medicine, Jawaharlal Nehru Medical College, Datta Meghe Institute of Higher Education and Research (Deemed to be University), Wardha, IND; 2 Anatomy, Jawaharlal Nehru Medical College, Datta Meghe Institute of Higher Education and Research (Deemed to be University), Wardha, IND; 3 Dermatology, Byramjee Jeejeebhoy (BJ) Medical College and Civil Hospital, Ahmedabad, IND

**Keywords:** stevens-johnson syndrome (sjs), toxic epidermal necrolysis (ten), drug eruptions, pharmacovigilance, prevention, management, diagnosis, classification, dermatological manifestations, cutaneous adverse drug reactions

## Abstract

Cutaneous adverse drug reactions (CADRs) are one of the most broadly studied and rigorously researched conditions in recent dermatological advancements. Also termed as "toxidermia," they are heavily involved and are of utmost importance to be understood and studied in the modern healthcare industry. In simple terms, they are dermatological manifestations which result from systemic drug administration to patients. Since allopathy is influenced by the medicines and drugs provided to the patients, cutaneous skin eruptions are a common occurrence in recent times. It is a need of the hour to understand the causative factors for such skin eruptions and the correct management and handling of such disorders to provide better healthcare to patients. The withdrawal of the causative drug which induces the reaction plays a key role in treatment. The risk factors are to be thoroughly studied, and dosages must be in accordance with the patient's situation. They are some of the common public health problems. The age group which is affected is highly variable as people from all age groups can be affected. Those who are affected comprise approximately 10% of all hospitalized patients, and it is also observed in about 1-4% of people who are on multiple medications.

## Introduction and background

Cutaneous adverse drug reactions (CADRs) are a common and significant complication associated with the use of various medications [1]. This review article provides a comprehensive overview of CADRs, including their classification, clinical scenarios and presentations, diagnosis, management, risk factors, prevention, and future research directions. Most CADRs have a relatively benign and non-problematic course, but some other rare drug reactions can also be severe and life-threatening. However, death is unlikely in the majority of the cases [2]. A thorough understanding of CADRs is very important for healthcare professionals to instantaneously recognize and manage these reactions, thereby minimizing patient morbidity and optimizing various treatment outcomes. It is also vital to highlight the importance of pharmacovigilance and adverse drug reaction reporting systems in identifying and preventing CADRs [3]. The main symptoms and common clinical findings include maculopapular eruptions, urticarial reactions, redness, itching, well-defined lesions, bullous reactions, erythroderma, discoloration, and ulcers [4]. CADRs refer to a spectrum of dermatological manifestations that arise as a result of drug therapy [5].

The classification of CADRs can be done on the basis of various types based on their immunological or non-immunological mechanisms. It is important to note that there must be an involvement in immunohistopathological examination of all CADRs to diagnose them and treat them accordingly [6]. The classification helps us to discuss the many different categories of CADRs, which include allergic reactions, hypersensitivity reactions, phototoxic reactions, photoallergic reactions, drug-induced pigmentation, urticarial reactions, pseudolymphomatous reactions, fixed drug eruptions, and severe reactions such as Stevens-Johnson syndrome (SJS) and toxic epidermal necrolysis (TEN) [7]. For example, anaphylaxis is a life-threatening condition, for which causative factors are certain drugs and medications. The discussion of the pathophysiology of CADRs dives into the underlying mechanisms of CADRs, focusing on immunological and non-immunological processes [8]. It explores type I and type IV hypersensitivity reactions, complement-mediated reactions, as well as phototoxic and photoallergic reactions [9]. Clinical presentations of CADRs show variable manifestations in many dermatological presentations; however, there are a few common areas in all clinical presentations of this disease. Some of the most common clinical manifestations observed in different types of CADRs including maculopapular eruptions, urticarial eruptions, numerous skin eruptions, bullous reactions, erythroderma, acute generalized pustulosis on the skin, SJS, and TEN are among the key presentations of cutaneous eruptions. However, most patients with CADRs do not need to be hospitalized because, at the onset, severe and life-threatening drug reactions must be taken into consideration and assessed accordingly [10]. The majority of clinical presentations are of the mild variety. Only a few variants are known to have caused death in patients.

## Review

Methodology

**Figure 1:**
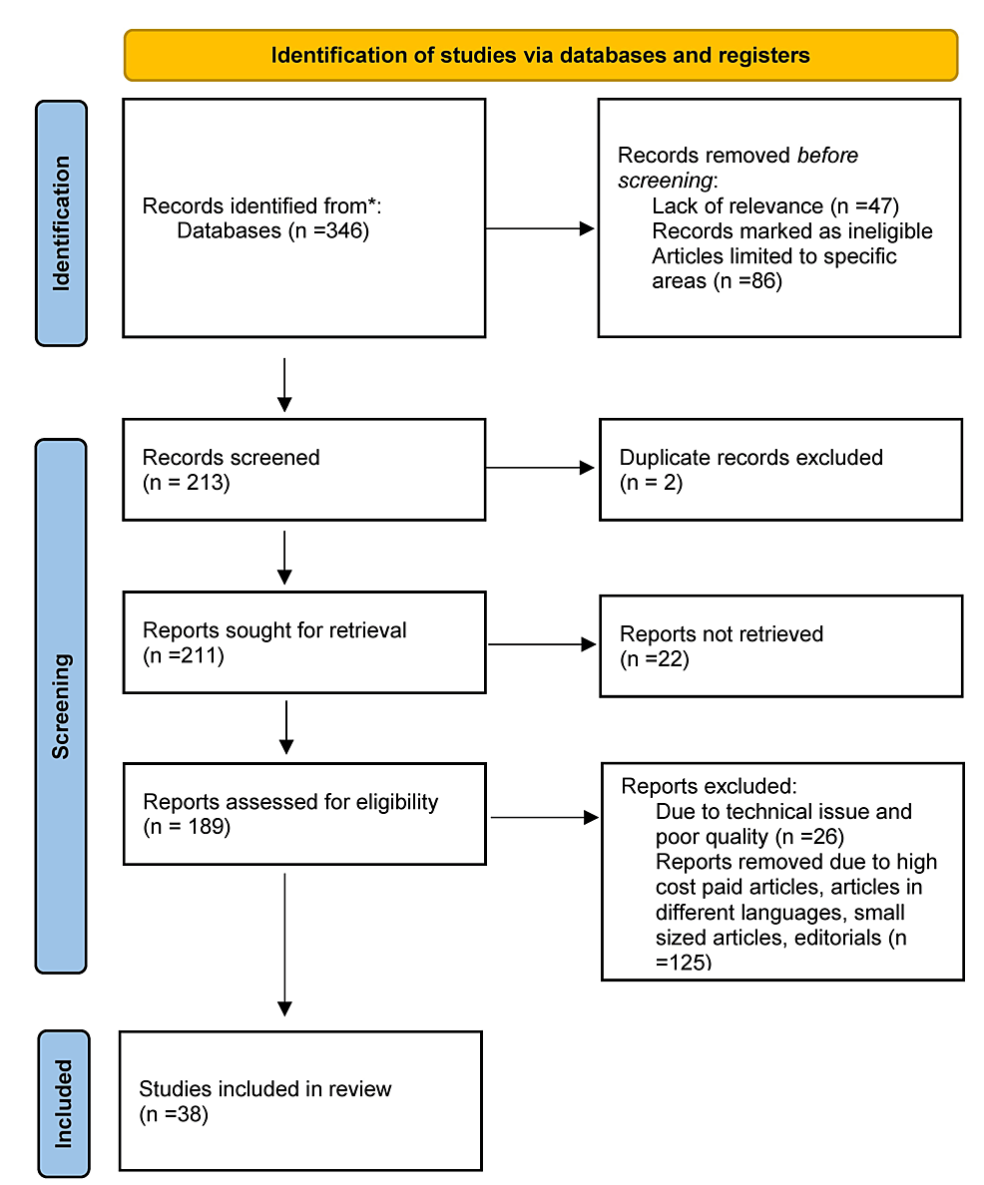
PRISMA methodology PRISMA: Preferred Reporting Items for Systematic Reviews and Meta-Analyses

Relevant PubMed articles were thoroughly scanned, and information was extracted in accordance with the subsequent topics. Multiple articles related to CADRs were chosen, filtered, and rejected as per relevance. Modern treatment modalities of the eruptions and diseases were also looked upon. Articles related to modern medicine having an influence on skin eruptions and their pathophysiology were also screened. The query terms were "cutaneous adverse drug reactions" OR "skin eruptions"; "treatment" OR "modalities"; and "diagnosis" OR "identification." Studies retrieved from these electronic searches and relevant references included in the bibliography of those studies were reviewed. Figure* *1 highlights the use of the Preferred Reporting Items for Systematic Reviews and Meta-Analyses (PRISMA) method in research methodology.

Accurate diagnosis of CADRs

The quick, efficient, and accurate diagnosis of CADRs is crucial for appropriate management and for the response and treatment of specific disorders [11]. It includes a lot of variables such as understanding and studying the numerous diagnostic approaches used in CADRs, including patient history and physical examination, patch testing, skin biopsy, laboratory investigations, and provocation tests. It emphasizes the importance of a multidisciplinary approach involving dermatologists and allergists for a comprehensive evaluation. It also should be noted that it is sometimes difficult to determine which drug is responsible for a particular skin eruption.

**Figure 2:**
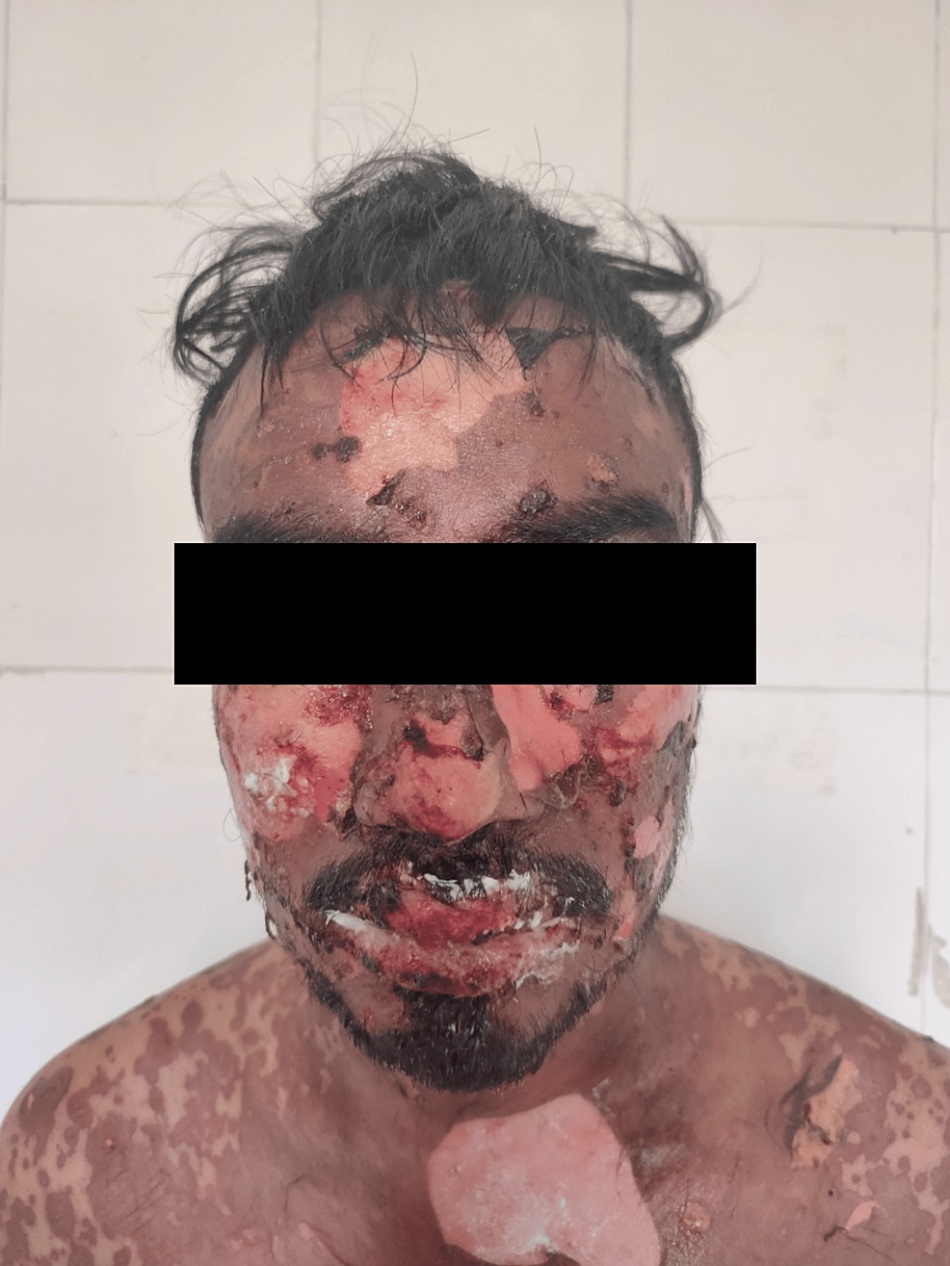
A typical presentation of toxic epidermal necrolysis Consent has been taken from the patient to use his clinical image in this article. Image Credit: Author

There can be various causes for a distinct skin lesion. The skin eruptions may progress rapidly and may also get infected in severe cases, leading to further complications. Sepsis is the leading cause of death in cases of secondary infection on skin lesions. It is also a common occurrence in SJS and TEN [12]. The skin lesions might be difficult to differentiate from viral exanthemas and some other skin diseases [13]. It is important to develop specific diagnostic markers to assess the seriousness and to have a predictable prognosis to provide improvised patient care. Some of the typical scenarios comprise rashes, abnormal hematological counts, fever, redness, itching, peeling of the skin, pigmentation, and also the involvement of visceral organs. An image of a case of TEN is depicted in Figure 2 [14]. The presence of lesions on the body's mucous membranes is also common. Tissue death or gangrene is the characteristic feature of TEN. On such skin lesions, herpes zoster may also infect the patient. Two of the common symptoms occurring in TEN and SJS are fever and malaise. Patients' initial complaint is fever due to an underlying infection or tissue death. Infection is a very common occurrence in patients with Stevens-Johnson syndrome. The skin has a potato-peel appearance in epidermal necrolysis, which is a typical clinical manifestation in the majority of patients. Purple-colored spots on the body, especially on the back and chest, are the hallmark features of cutaneous manifestations of epidermal necrolysis [15]. It is commonly caused by antibiotics and anticonvulsants such as phenytoin and lamotrigine. Among anticonvulsants, carbamazepine is also known to be the culprit drug in some cases. Bullous eruptions present as blisters on the skin. Folliculitis is also observed in drug reactions.

Urticaria is also a common occurrence among cutaneous eruptions. Topical application or ingestion of penicillin does cause urticaria in some cases. Non-steroidal anti-inflammatory agents (NSAIDs) in general are reportedly known to have caused urticaria in many cases. Raised, swollen, and fleshy bumps on the skin are hallmark features of urticaria. NSAIDs also cause lichenoid skin eruptions, exanthematous reactions, and photosensitivity reactions. Psoriasis presents as dry and itchy patches, covered with micaceous scales on the skin.

All the mentioned differential diagnoses are the result of informative and efficient history-taking in the majority of the cases. This requires awareness and knowledge about the causative drugs leading to skin reactions. In many cases, a common pattern is found that the symptoms of the drug reaction start appearing after a week of taking the causative drug. In some cases, symptoms may develop as early as four days or may even begin to appear at the beginning of the fourth week. When trying to determine the culprit drug, especially in patients taking multiple medications, the determination of the drug that was prescribed most recently is essential. The newest drug is more likely to be the cause, even if they have only been taking it for two or three months.

Management and treatment of CADRs

There are several variables to be considered for the management of CADRs [16]. The effective management techniques for CADRs involve discontinuation of the culprit drug, treatment in consideration of the patient's needs, topical and systemic interventions, and specifically designed approaches for severe reactions. The treatment modalities must be precisely adjusted for patients who take multiple medications or those who are taking multi-drug therapy [17]. It is important to have a set guideline for general measures and specific treatment modalities based on the type and severity of CADRs. As there are innumerable amount of prognoses and types of CADRs, the management and patient-specific treatment vary from person to person. For example, there is always a fixed treatment method for patients with epidermal necrolysis [18]. The treatment always starts with stopping the use of the drug causing the reaction [19].

There is an immediate response in some cases as soon as there is cessation of the causative drug. Even though the drug has been withdrawn, the symptoms caused by it need to be managed by therapeutic drug intervention. This involves the provision of pain medications, antibiotics to cure infection caused by the skin lesion, and anti-inflammatory drugs in cases of edema and urticaria. Anti-pyretic drugs are provided to manage fever. Nutrition is also essential as the skin needs nourishment to repair the damage to the lesion. Protein and other nutrients are administered intravenously in patients with epidermal necrolysis.

One of the most important tools which is used in managing and assessing the severity of the reaction is the Severity-of-Illness Score for Toxic Epidermal Necrolysis (SCORTEN). This method has been used for more than 20 years to predict the prognosis and measure the severity of the disease in patients [20]. An accurate estimation is important for the classification of the type of CADR and also for the prognosis to provide patient-specific treatment modality depending upon the severity of the drug reaction [21]. Medications used for the treatment of SJS are corticosteroids. Pain medication is provided to the patient to reduce the severity. Intravenous immunoglobulins have also been given in many cases of SJS. Replacing fluids and providing nutrients are of utmost importance for the rebuilding of the skin due to TEN. For eye care, eye drops and ointments are used to prevent further damage and heal the mucus membranes.

The coronavirus disease 2019 (COVID-19) has also contributed to managing skin eruptions caused due to vaccination [22]. Drug-induced psoriasis also has specific treatment methods. Anti-malarial drugs and anti-hypertensive drugs are known to have caused psoriasis. In the case of psoriasis, little to no improvement is seen upon the cessation of the causative drug. Additional dermatological therapy is often required to combat drug-induced psoriasis. Corticosteroids and vitamin D supplements are often recommended for psoriasis treatment [23]. Generalized bullous fixed drug eruptions (GBFDE) appear as blisters and erosions on the skin. They can be treated by oral prednisolone. Steroid therapy is also quite effective after the patient has stopped taking the drug causing the drug eruption [24]. Cyclosporine has also been used in some rare cases to cure GBFDE [25]. In one specific case, stoppage of further blistering was observed after 24 hours post-administration of cyclosporine [26]. Photosensitivity reactions are also observed in patients who were given doxycycline and tetracycline. Anti-cancer drugs also have a history of causing reactions. The drugs prescribed for colorectal cancer, lung cancer, head and neck cancer, and pancreatic cancers may cause rashes on the upper body and the face. Cancer therapy drugs, which are known to cause necrolysis, can be substituted according to the patient's immune response, and afterwards, gradual response and betterment can be observed after cancer therapy has stopped [27,28]. Some cancer drugs causing skin eruptions are gefitinib and cetuximab. They are known to cause folliculitis as they inhibit the epidermal growth factor receptor (EGFR). Anti-viral drugs, upon administration, have shown a response to viral skin eruptions [29]. Urticaria treatment methods involve the use of anti-inflammatory drugs such as cetirizine, loratadine, and fexofenadine. A summarised table providing the information for the management of CADRs is given in Table 1.

**Table 1:** Summary of the causative drugs, the drug reaction, and their management NSAIDs: non-steroidal anti-inflammatory drugs; GBFDE: generalized bullous fixed drug eruptions Table Credit: Author

Causative drug	Clinical manifestation	Management
Antibiotics (tetracycline and sulfonamides) and anticonvulsants	Stevens-Johnson syndrome and toxic epidermal necrolysis	Corticosteroids, cyclosporine, IV immunoglobulin G, and fluids
Anti-malarial and anti-hypertensive drugs	Psoriasis and lichenoid rash	Topical corticosteroids and vitamin D supplements
Chemotherapy drugs (gefitinib, cetuximab)	Rashes, pustules, and necrosis	Cessation/reduction of the drug, antibiotic-containing creams, and corticosteroids (fluocinonide, clobetasone)
Antibiotics (penicillin) and NSAIDs	Urticaria and anaphylaxis	Anti-histamine drugs (cetirizine, loratadine, fexofenadine) and systemic corticosteroids
Antibiotics (rifampicin, metronidazole), ibuprofen, and paracetamol	GBFDE	Oral prednisolone
NSAIDs, thiazide diuretics, and contraceptives	Photosensitivity	Photo-protection and topical corticosteroids

Risk factors contributing to CADRs

A lot of factors contribute towards the causation of fixed drug reactions. Many variables must be taken into consideration to assess risk factors which cause CADRs such as individual susceptibility, genetical predisposition, and drug-related factors that influence the occurrence and severity of these reactions. Results derived from hospital-based populations suggest and conclude that all the fixed drug eruptions had no known cause and were unexpected [30]. In France, a dermatologically controlled study stated that the majority of patients developed exanthematous reactions, whereas only a small minority of patients developed erythroderma and SJS/TEN [31]. Out of all the drugs, the primary group of drugs which pose a risk factor causing CADRs are antimicrobials, anti-epileptic drugs, and NSAIDs [32]. Females are also at a higher risk of having CADRs as shown by a 2005 study. There is also an increased severity in the manifestations of skin eruptions in women [33]. Age is not a relevant factor, but people belonging to older age groups tend to have a higher susceptibility to developing CADRs as they are exposed to many medications due to ageing [34]. Drug allergy is indeed found to be lower in children, but infants in particular are prone to cutaneous eruptions as their body's capacity to fully metabolize drugs has not been fully developed yet [35]. Other causative factors include having a history of food allergies or common illnesses such as hay fever. Such individuals are at a higher risk of developing such reactions. Also, increased exposure to a particular drug due to high doses, repeated doses, or prolonged use may also contribute to the development of skin eruptions.

Prevention of CADRs

Prevention plays an extremely vital role in reducing the severity and burden of CADRs. There are some basic steps involved in preventing drug reactions. This involves the identification of the subgroup of patients who are likely to be susceptible to the adverse effects and modifying the treatment choice accordingly. We must ensure that the treatment plan will mitigate all problems without causing subsequent damage and causing skin eruptions. Preventive measures can be implemented and improved upon through patient education, spreading awareness, surveillance and data analysis, alternative medications for patients, and the newly emerging role of pharmacogenetic testing in minimalizing the risk of CADRs. Pharmacogenetic testing eliminates all causative drugs leading to skin eruptions. However, it is not widely available and is not cost-effective in many regions. If made more extensive and inclusive, while being cost-effective, such testing would significantly reduce the occurrence of such diseases. The consumption of drugs causing drug eruptions should be monitored and checked in accordance with the patient's history. Penicillin and NSAIDs are also major causes of CADRs, and they must be avoided in specific cases in order to prevent drug reactions from occurring [36].

In the majority of cases, there is no way to know how a patient will react to a medication. This is where proper management, history-taking, and awareness about medications causing skin eruptions come into action. The patient should also know about the drugs that have caused a reaction in the past and should refrain from taking similar medications. An adequate understanding of a patient's medication usage is necessary. People should inform their doctor about any prior history and about the medications they are currently taking.

Pharmacovigilance and reporting systems

Pharmacovigilance is the method of monitoring the effects of medicines and drugs in order to identify, evaluate, and rectify drug reactions in patients. Pharmacovigilance programs and adverse drug reaction reporting systems are essential for identifying and monitoring CADRs. There should be a heavy emphasis on the importance of robust reporting mechanisms and the role of healthcare professionals in reporting various suspected drug reactions. Pharmacovigilance must be promoted in the highly evolving and developing healthcare sector so as to be cautious and to avoid CADRs. Having pre-requisite knowledge and proper history-taking is a must to avoid such diseases, and there must be an instantaneous implementation of reporting systems to counter CADRs. Awareness must be spread about pharmacovigilance in the society to prevent drug reactions [37]. The most common method to detect such drug reactions is relying on spontaneous reports. However, the massive disadvantage is that the low reporting rate of spontaneous reports is a serious limitation of pharmacovigilance.

Future development, research perspectives, and treatment modalities of CADRs

Further advancements in the study of CADRs are necessary for the betterment of the healthcare sector and to provide optimum patient care. Future development helps in minimizing the occurrence of CADRs and other drug-induced diseases. It is also important to highlight the need for ongoing research to improve diagnostic techniques, develop personalized medicine approaches, identify risk factors, and enhance pharmacovigilance efforts in CADRs [38]. The future development in enhancing pharmacovigilance efforts involves extensive testing, making drug therapy cost-effective, and the patients should be tested prior to treatment in order to reduce the risks of skin eruptions. The risk factors can only be minimized when there is awareness and the doctors as well as patients have adequate knowledge. Further advancements in research will reduce the severity and occurrence and prevent CADRs.

## Conclusions

This research article provided us with a thorough understanding of CADRs, with emphasis on the classification, causative factors, clinical presentations, and diagnosis. It gave us a better understanding to provide reaction-specific treatment and prophylaxis in susceptible patients with past or family history of CADRs. Patients receiving multiple medications for comorbidities should be closely monitored for any kind of CADRs to provide early management. Provision of facilities for pharmacogenetics also proves to be vital in patient management.
